# Development of CS/PLA Composites with Enhanced Ductility via PBS Elastomer Reinforcement

**DOI:** 10.3390/ijms26104643

**Published:** 2025-05-13

**Authors:** Tingqiang Yan, Kang Chen, Xiaodong Wang, Yingjie Qiao

**Affiliations:** 1 College of Material Science and Chemical Engineering, Harbin Engineering University, Harbin 150001, China; yantingqiangheu@163.com; 2 Key Laboratory of Bio-Based Material Science & Technology, Ministry of Education, Northeast Forestry University, Harbin 150040, China; chenk0777@nefu.edu.cn

**Keywords:** polylactic acid, chitosan, PBS, high ductility

## Abstract

Polylactic acid (PLA) exhibits remarkable biocompatibility and biodegradability, rendering it a highly promising material for applications in packaging and disposable products. However, its inherent brittleness, low melt strength, and slow crystallization rate significantly restrict its practical uses. Our previous studies have shown that incorporating the ADR chain extender can yield chitosan–polylactic acid–ADR (CS/PLA-ADR) composites with outstanding antibacterial properties, enhanced biodegradability, and the capability to effectively block water vapor and oxygen. However, the low elongation at break (less than 10%) limits its application in scenarios that require high ductility. To enhance the toughness of the CS/PLA-ADR composites, the flexible biodegradable polybutylene succinate (PBS) is innovatively introduced. The mechanical properties of PBS can be compared with polyethylene and polypropylene, providing high strength and toughness. The mechanism of introducing PBS is to construct a good, toughened structure through the flexible structure of PBS in collaboration with ADR toughening agent, achieving a balance between strength and toughness in CS/PLA-ADR-PBS composites. The incorporation of PBS is anticipated to improve the ductility of CS/PLA-ADR composites. This study systematically investigates the effects of varying PBS content (0–30%) on the properties of CS/PLA-ADR-PBS composites, aiming to determine the optimal PBS content and elucidate the mechanism by which PBS enhances the overall performance of the composites. The results indicate that when the PBS content is 20%, the composites exhibit optimal overall properties. This research provides a theoretical foundation and technical support for the development of environmentally friendly and sustainable packaging materials, offering significant research value and broad application prospects.

## 1. Introduction

Increasing concerns about the depletion of fossil resources and the significant environmental issues linked to the use of fossil-based plastics have strongly propelled the development of a sustainable circular plastic economy. The production of conventional and biodegradable plastics from bio-based materials has emerged as a specialized solution within this circular plastic system [1]. In the packaging sector, particularly for food and beverage bags and containers, renewable resource-based plastic materials can effectively reduce dependence on traditional fossil fuels and significantly lower the risk of long-term environmental pollution caused by plastic products after their use [2].

Polylactic acid (PLA) is derived from renewable resources that are rich in starch, cellulose, or sugars, such as corn, cassava, sugarcane, sugar beet, and straw. It has garnered significant attention due to its excellent biocompatibility and biodegradability [3]. Additionally, PLA exhibits outstanding mechanical properties, with strength and stiffness comparable to those of petroleum-based polymers like polystyrene (PS) and polyethylene terephthalate (PET) [4]. However, the poor toughness, low melt strength, and low crystallinity of PLA limit its application in various fields. These drawbacks can be addressed through physical and chemical modification methods.

The primary methods of physical modification include blending with plasticizers, nucleating agents [5], and inorganic fillers [6], and mixing with other tough materials [7]. Chitosan (CS) is a good biodegradable material based on biological materials. Its main components are derived from shrimp shells or crab shells and it is an excellent filler for PLA. Zhu et al. found that the composite made by modifying CS and combining it with PLA has excellent antibacterial and flame-retardant properties. Meanwhile, the tensile strength of the CS/PLA composite also increased by 8.9% [8]. Chemical modification can also be achieved by adding chain extenders, such as poly (styrene-acrylic-co-methacrylate glycidyl) copolymer (ADR), to lengthen the molecular chains. Mateusz et al. significantly enhanced the impact strength and toughness of waste PLA by adding ADR, and at the same time, the glass transition temperature and thermal stability of the material were also improved to a certain extent [9]. In practical applications, a combination of physical and chemical modifications is often employed to optimize material properties. Our previous research indicates that the cycloaddition opening reaction of the ADR chain extender can enhance the interfacial compatibility of CS/PLA composites, resulting in the preparation of thermodynamically compatible CS/PLA-ADR composite films [10].

ADR facilitates the connection of terminal groups in polylactic acid molecular chains through ring-opening chain extension. Additionally, ADR can link polylactic acid and CS through ring opening, thereby enhancing compatibility. The resulting CS/PLA-ADR composite films exhibit excellent antibacterial properties and biodegradability, and the ability to block water vapor and oxygen. However, these composite films have a relatively low elongation at break (<10%), which somewhat limits their application in scenarios requiring high ductility.

As a typical biopolymer, polybutylene succinate (PBS) not only possesses excellent mechanical properties but also demonstrates good processability and complete degradability [11]. Compared to other biodegradable plastics, the mechanical properties of PBS are comparable to those of polyethylene and polypropylene, providing high strength and toughness. Blending PBS with PLA can improve the ductility of PLA without compromising its biodegradability [12], and the desired characteristics of the composite material can be achieved by adjusting the blending ratio [13]. For instance, Sakaowduen et al. [14] prepared blends of PBS and PLA using an extrusion molding process and systematically investigated the mechanical properties of the composites with varying PBS content. The results of the study indicated that incorporating an optimal amount of highly flexible PBS into the PLA matrix can significantly improve the impact strength, elongation at break, crystallinity, and thermal stability of the PBS/PLA composites [15].

Based on the background and previous research findings, this study investigated the effects of varying levels of PBS addition on the mechanical properties, hydrophilicity, crystallization behavior, thermo-mechanical properties, and rheological properties of CS/PLA-ADR composites. The objective was to improve the ductility of CS/PLA-ADR composites and broaden their application fields. By comparing these composites with those that do not contain PBS, the optimal level of PBS addition was identified, and the mechanisms through which PBS enhances the overall performance of CS/PLA-ADR composites were summarized.

## 2. Results and Discussion

### 2.1. Effect of PBS Contents on Chemical Properties of CS/PLA-ADR-PBS Composites

**Figure 1:**
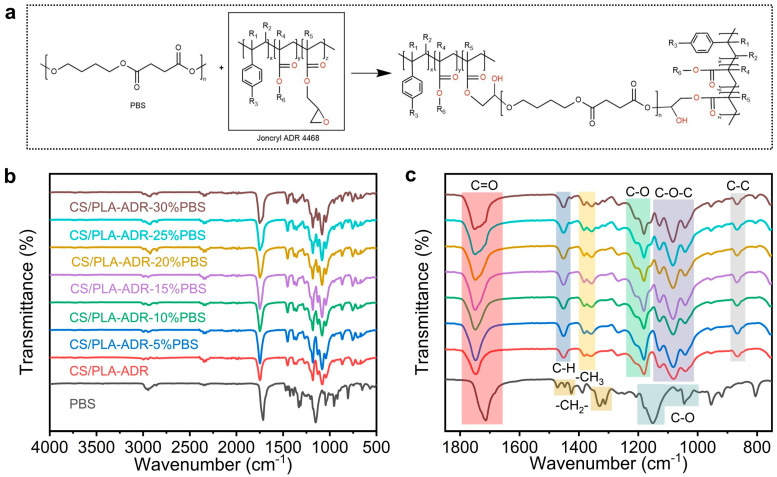
(**a**) The chemical reaction equation of PBS and ADR, (**b**) the FTIR spectra of CS/PLA-ADR-PBS composites with different PBS contents, and (**c**) the enlarged FTIR spectrum.

Figure 1a illustrates the reaction equation between PBS and ADR. In the high-temperature environment of melt extrusion for the composite, the carboxyl and hydroxyl end groups of PBS can undergo a nucleophilic ring-opening reaction with the epoxy groups in the ADR molecular chain, resulting in chain extension. Figure 1b,c present the FTIR spectra of the composite material. In the infrared spectrum of PBS, the absorption peaks at 2944 cm^−1^ and 2856 cm^−1^ are attributed to the asymmetric and symmetric stretching vibrations of the -CH_2_- groups in the PBS main chain, respectively. The strong absorption peak at 1712 cm^−1^ corresponds to the stretching vibration of the ester C=O group. This peak is lower than the carbonyl absorption peak at 1750 cm^−1^ in PLA, indicating the flexibility of the PBS molecular chain and the influence of substituents on the vibration frequency. The absorption peaks at 1471 cm^−1^ and 1427 cm^−1^ are primarily associated with the scissoring and bending vibrations of the -(CH_2_)- groups. The absorption peak at 1388 cm^−1^ may be attributed to the wagging vibration of the -(CH_2_)- groups or the bending vibration of terminal -CH_3_ groups. The absorptions in the region of 1332 cm^−1^ and 1311 cm^−1^ reflect vibrational modes that couple C-H bending and C-O stretching vibrations. The peaks at 1147 cm^−1^ and 1045 cm^−1^ are mainly attributed to the stretching vibrations of C-O–C and C-O bonds. Additionally, the absorption peaks at 954 cm^−1^, 917 cm^−1^, and 806 cm^−1^ are related to the skeletal vibrations or out-of-plane vibrations of the -(CH_2_)- groups in the PBS main chain [16].

In the FTIR spectrum of the CS/PLA-ADR-PBS composite, the ester C=O absorption peak at 1751 cm^−1^ primarily originates from PLA. This peak is slightly higher than the ester C=O absorption peak in PBS, which occurs at 1712 cm^−1^. This difference indicates that the carbonyl absorption of PLA remains dominant in the composite, suggesting a degree of separation between the carbonyl absorption peaks of PLA and PBS. Additionally, the absorption peak at 1450 cm^−1^ corresponds to the scissoring or bending vibrations of the -CH_2_/-CH_3_ groups present in both PLA and PBS. The absorption peak at 1357 cm^−1^ is primarily attributed to the bending vibrations of the -CH_3_ groups in the PLA side chains. In the regions of 1180 cm^−1^, 1130 cm^−1^, 1083 cm^−1^, and 1039 cm^−1^, the C-O-C and C-O stretching vibrations of PLA, PBS, and CS overlap and couple. The intensity of the absorption peaks in these regions is enhanced, and slight peak shifts may occur. This phenomenon indicates an enhancement of intermolecular hydrogen bonding and polar interactions within the composite [17].

Additionally, the absorption peaks at 863 cm^−1^ and 752 cm^−1^ are primarily attributed to the out-of-plane bending vibrations of aromatic ring C-H bonds, indicating the presence of benzene ring structures in ADR. As the content of PBS increases, a shoulder peak emerges near 1727 cm^−1^, adjacent to the PLA carbonyl absorption peak at 1750 cm^−1^. This phenomenon is attributed to the enhanced ester C=O absorption of PBS, which overlaps with the carbonyl absorption of PLA [18]. The C-O stretching vibration peak at 1081 cm^−1^ exhibits a slight red shift, accompanied by an increase in peak intensity. Furthermore, the heightened intensity of absorption peaks at 1180 cm^−1^, 1041 cm^−1^, and 954 cm^−1^, along with the emergence of a new absorption peak at 920 cm⁻¹, suggests that the increase in PBS content not only gradually reveals its own absorption characteristics but also modulates the intermolecular interactions among PLA, CS, and ADR through hydrogen bonding, polar coupling, and alterations in local crystallinity and aggregation state. This indicates that the epoxy group in ADR undergoes a chemical reaction with the carboxyl group in the PLA and PBS molecular chain (Figure 1a), increasing the molecular weight of PLA and PBS. It thereby enhances various properties of the CS/PLA-ADR-PBS composites [19]. The same phenomenon has also been confirmed in other studies.These changes lead to new vibrational features and variations in peak shape and position within the composite material, ultimately enhancing the overall compatibility and structural performance of the composite [20].

### 2.2. Mechanical Properties of Composites

**Figure 2:**
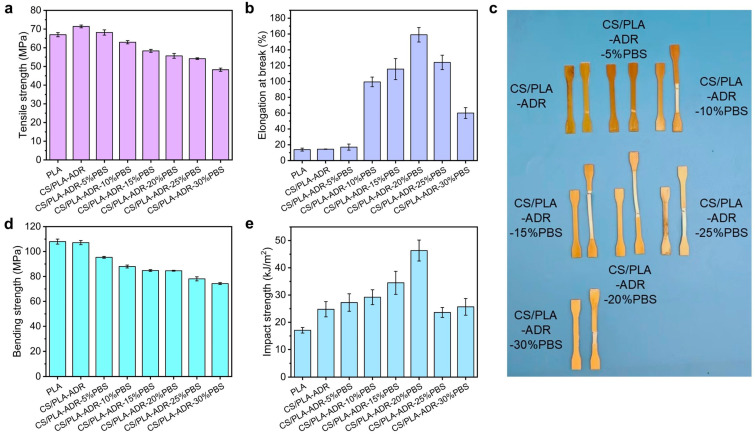
The (**a**) tensile strength, (**b**) elongation at break, (**c**) comparison images before and after stretching, (**d**) bending strength, and (**e**) impact strength of CS/PLA-ADR-PBS composites with different PBS contents.

Figure 2a illustrates the trend in tensile strength of the composite with increasing PBS content. The results indicate that the tensile strength of the composite gradually decreases as the PBS content increases. Specifically, the tensile strength of the CS/PLA-ADR composite material is 71.40 MPa when no PBS is present. When the PBS content reaches 30%, the tensile strength of the CS/PLA-ADR composite material significantly decreases to 48.25 MPa. This reduction is attributed to PBS being a flexible polymer with inherently low tensile strength. We tested the tensile strength of pure PBS, which showed it to be 31.92 MPa and the tensile elongation at break to be 326.13%. Therefore, as the PBS content increases, the overall tensile strength of the composite material decreases significantly [21].

The variation in elongation at break of the composite material with varying PBS content is illustrated in Figure 2b. As the PBS content increases, the elongation at break of the composite material initially rises and then declines. The photographs of the samples taken before and after tensile strength testing, presented in Figure 2c, align with the observed trend in elongation at break. When the PBS content reaches 20%, the elongation at break of the composite material attains a maximum value of 159.02%. This phenomenon can be attributed to the influence of the chain extender ADR, which facilitates the formation of a cross-linked network structure among CS, PLA, and PBS. The incorporation of PBS into the composite system introduces highly flexible molecular chains. At low PBS concentrations, as the amount of PBS increases, the composite material demonstrates enhanced plastic flow during tensile testing, resulting in a gradual increase in elongation at break. However, because the molecular weight of PBS is lower than that of PLA, excessive PBS content leads to a higher concentration of hydroxyl and carboxyl groups within the composite. This situation hampers the ability of ADR to form sufficient cross-linked structures. As a result, the interfacial compatibility of the composite deteriorates, limiting the potential for increased plastic flow and adversely affecting the elongation at break of the composite.

Figure 2d illustrates the variation in bending strength of the composite material with different PBS contents. The results indicate that as the PBS content increases, the bending strength of the composite shows a decreasing trend, which aligns with the trend observed in tensile strength. When the PBS content is 0%, the bending strength of the CS/PLA-ADR is 107.15 MPa. As the PBS content rises to 10%, the bending strength decreases to 87.99 MPa; at 20% PBS content, it further declines to 84.59 MPa; and when the PBS content reaches 30%, the bending strength drops to 74.30 MPa. This phenomenon can be attributed to the introduction of PBS, which increases the number of flexible molecular chains in the composite system, thereby reducing the bending strength of the material. The main biodegradable materials used for toughening PLA also include poly(butylene adipate-co-terephthalate) (PBAT). The comparison of the strength and elongation at break of PLA/PBAT composites with carbon nanotubes or starch as fillers indicates that the toughening effect of PBS is on par with that of PBAT, and they both have excellent toughening effects on PLA composite [22,23].

As shown in Figure 2e the impact strength of the composite initially increases and then decreases with rising PBS content. At lower levels of PBS, the chain extender ADR effectively facilitates the formation of a cross-linked network structure within the composite. During this phase, stable interfacial layers can develop between CS and PLA, CS and PBS, as well as PBS and PLA. The flexible polymer PBS serves as a toughening agent, significantly enhancing the impact strength of the CS/PLA-ADR-PBS. However, when the PBS content becomes excessively high, the interfacial compatibility of the composite deteriorates. Under external impact forces, the interfacial layers are susceptible to debonding, resulting in the formation of larger defects that directly compromise the integrity of the composite and subsequently affect its toughness.

**Figure 3:**
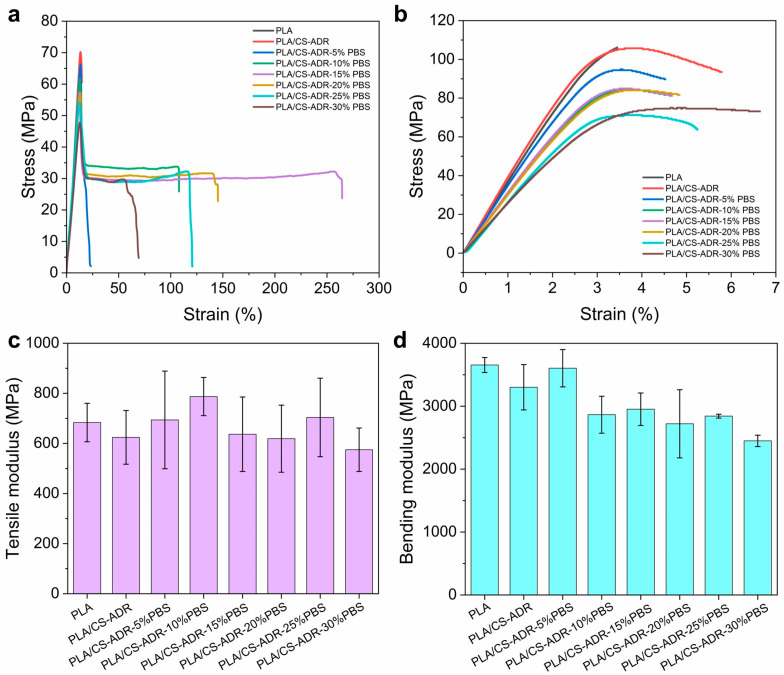
The (**a**) stress–strain curves of tensile strength, (**b**) stress–strain curves of bending strength, (**c**) tensile modulus, and (**d**) bending modulus of CS/PLA-ADR-PBS composites with different PBS contents.

Figure 3 shows the stress–strain curves and various modulus data of CS/PLA composites after adding different contents of PBS. The conclusion was the same as that obtained for tensile strength and elongation at break. After adding 15% and 20% PBS, the composites with the best comprehensive performance of tensile strength and toughness are obtained (Figure 3a). It can be seen from the stress–strain curve of the bending strength test that with the addition of PBS, the toughness of the composite increases significantly, and the bending stress–strain curve also begins to show a yield phenomenon (Figure 3b). In addition, tensile modulus and bending modulus are also important parameters for evaluating the performance of composite materials. As shown in Figure 3c, the tensile modulus of the composites slightly increases after the addition of PBS. This is mainly due to the certain improvement in the elongation at break of the composite material after the addition of PBS. As shown in Figure 3d, the bending modulus of the composite material decreased significantly after the addition of PBS, which indicates that the toughness of the composite material was enhanced to a certain extent, suggesting that the addition of PBS achieved the purpose of toughening the PLA/CS composite.

### 2.3. The Effect of PBS Contents on the Morphology of CS/PLA-ADR-PBS Composites

**Figure 4:**
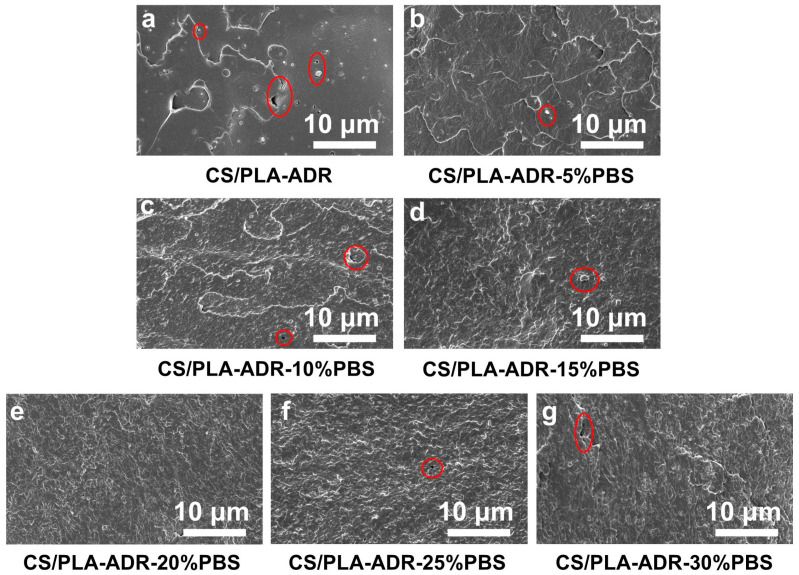
SEM images of sample sections of CS/PLA-ADR-PBS composites with different PBS contents were obtained by brittle fracture in liquid nitrogen.

Figure 4a–g illustrate the microstructure of the fracture surfaces of PLA/PBS/CS materials with varying PBS content, obtained through brittle fracture in liquid nitrogen.

In the absence of PBS, the material surface appears relatively smooth, with small particles embedded within it. As PBS is introduced, the surface of the material becomes increasingly rough. This change is attributed to the development of a co-continuous phase structure between PLA and PBS. The high toughness of PBS contributes to the formation of this structure, which enhances the elongation at break of the blend [24]. With the increased concentration of PBS content, PBS droplets become distinctly distributed within the PLA matrix. At higher PBS concentrations, these droplets begin to aggregate, resulting in phase separation and the formation of an island–sea structure [25]. This phenomenon occurs due to the thermodynamic incompatibility and high interfacial tension between PLA and PBS [26]. When the PBS content is at 20%, the interface between the two phases is relatively indistinct, with PBS being more uniformly dispersed within the PLA matrix. The interfacial adhesion is stronger at this stage, resulting in enhanced toughness of the material.

**Figure 5:**
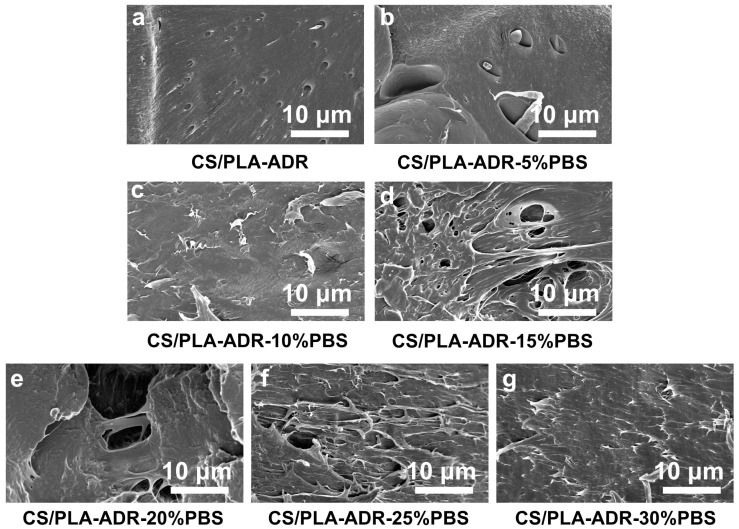
SEM images of the fracture surfaces of CS/PLA-ADR-PBS composites with different PBS contents after tensile testing.

To further investigate the morphology changes of the composite under tensile stress, SEM technology was employed to observe the fracture surfaces of CS/PLA-ADR-PBS composite samples following tensile testing, as illustrated in Figure 5a–g.

Figure 5a displays the tensile fracture surface of the CS/PLA-ADR composite without PBS, which appears relatively uniform and smooth. However, with the introduction of PBS, the surface morphology of the composite becomes progressively rougher and exhibits a torn fibrous structure. At a PBS content of 20% (Figure 5e), this torn fibrous structure is most pronounced, indicating that the composite absorbs more energy during tensile deformation, thereby achieving optimal toughness [27]. However, as the PBS content increases further, the torn structure gradually diminishes. This phenomenon occurs due to the onset of phase aggregation in PBS, which reduces the adhesion between the two phases. As a result, fractures are more likely to occur at the phase interfaces, and stress is less effectively transferred between the phases, leading to a decline in the toughening effect of the composite [28].

### 2.4. Effect of PBS Contents on Water Absorption of CS/PLA-ADR-PBS Composites

**Figure 6:**
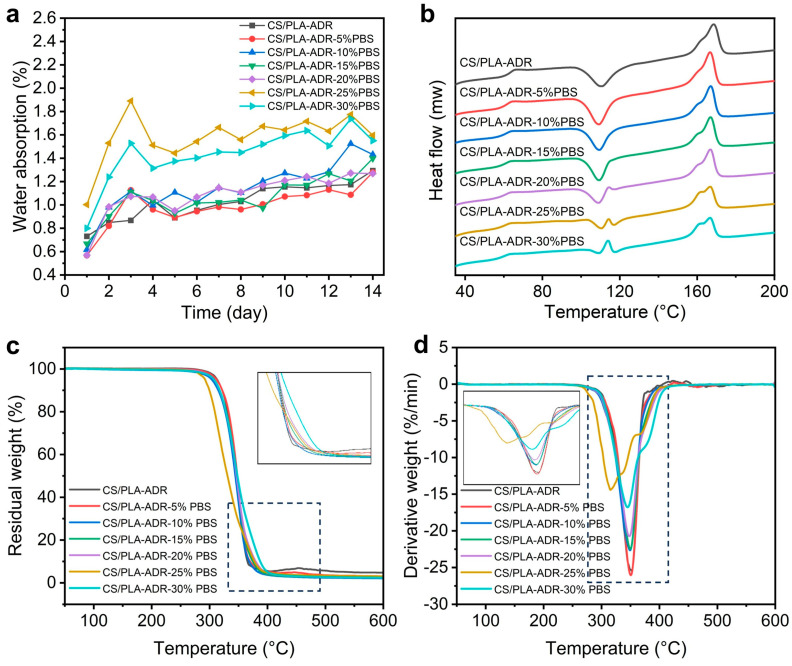
The (**a**) water absorption rate curve, (**b**) DSC curve, (**c**) TG curve, and (**d**) DTG curve of CS/PLA-ADR-PBS composites with different PBS contents.

Figure 6a illustrates the change in water absorption of the CS/PLA-ADR composite with the addition of PBS. As shown in the figure, when the PBS content is below 20%, the water absorption of the material remains essentially unchanged compared to when no PBS is added. This is because both PBS and PLA are hydrophobic materials, and the presence of the chain extender ADR enhances the bonding between PLA and PBS, thereby preventing significant phase separation. Consequently, the addition of PBS does not result in a noticeable change in the material’s water absorption. However, when the PBS content exceeds 25%, the water absorption of the material increases significantly. This is primarily because, with the increased addition of PBS, ADR can no longer effectively facilitate the bonding between PBS and PLA, resulting in reduced compatibility between the two. Consequently, slight phase separation occurs. This phase separation leads to the formation of phase interfaces within the material, making it easier for water molecules to penetrate the interior of the material, thereby increasing the water absorption rate.

### 2.5. Effect of PBS Contents on Thermal Properties of CS/PLA-ADR-PBS Composites

Figure 6b presents the DSC curves of the PLA/CS/ADR/PBS composite, with the corresponding thermodynamic data listed in Table 1. As the PBS content increases, the glass transition temperature (T_g_), cold crystallization temperature (T_cc_), and melting temperature (T_m_) of the composite all exhibit a decreasing trend, while the crystallinity gradually increases. Specifically, when the PBS content increases from 0% to 30%, the Tg of the composite decreases from 63.63 °C to 60.26 °C. The continuous decrease in T_g_ indicates that PBS, as a flexible polyester, is partially compatible with the PLA matrix, reflecting enhanced compatibility. This compatibility, through a plasticizing effect, improves the mobility of the PLA chain segments [28]. The reduction in T_g_ and T_cc_ indicates an improvement in interfacial bonding strength, thereby enhancing compatibility [29]. An exothermic peak is observed in the cold crystallization region of composites with high PBS content (20–30%). Since the melting point of PBS is approximately 114 °C, this exothermic peak can be attributed to the melting of PBS. This phenomenon results from the overlap of the recrystallization exotherm of PLA and the melting endotherm of PBS, suggesting that phase separation occurs within this temperature range [30]. The TGA curve of the CS/PLA-ADR-25%PBS composites has a certain deviation compared with the TGA curves of other proportions. This is mainly due to the situation of partial uneven mixing of the granular materials during the twin-screw blending process, and the agglomeration of CS causes a certain deviation in the TGA curve.

When the PBS content varies from 0% to 15%, the change in crystallinity is relatively minor, decreasing from 3.39% to 1.12% before rising again to 3.36%. This reduction in crystallinity is associated with the branching reaction between ADR and the CS/PLA-PBS mixture, which disrupts the structural regularity of the molecular chains. As the PBS content continues to increase, the crystallinity sharply rises from 3.36% to 9.93%. This is because, at higher PBS content, the compatibility between PBS and PLA in the composite weakens, causing PBS to form a continuous phase that releases the restrictions on chain segment mobility. Additionally, PBS crystallizes before PLA, and the resulting nuclei can serve as sites for the heterogeneous crystallization of PLA, thereby increasing its crystallinity. The distinct presence of a melting double peak further confirms that the composite material exhibits two different chemical structures at this stage.

### 2.6. Effect of PBS Contents on Thermal Stability of CS/PLA-ADR-PBS Composites

As shown in Figure 6c,d, when varying amounts of PBS are added to the CS/PLA-ADR system, the thermal decomposition behavior exhibits distinct quantitative changes. Within the range of 0% to 20% PBS addition, the samples display a single decomposition peak, with the peak temperature gradually decreasing as the PBS content increases—from 352 °C at 0% to 347 °C at 20% PBS. Concurrently, the maximum decomposition rate declines from −25.45%/min to approximately −20.68%/min. This trend indicates that the lower initial decomposition temperature of PBS (around 300 °C) causes the onset decomposition temperature of PLA to occur earlier, resulting in a reduction in overall thermal stability [31].

When the PBS content increases to 25% and 30%, the TGA and DTG curves exhibit a multi-peak phenomenon. Specifically, the CS/PLA-ADR-25% PBS sample displays three decomposition peaks at 315 °C, 338 °C, and 368 °C, while the 30% PBS sample shows two decomposition peaks at 346 °C and 377 °C. The low-temperature peak corresponds to the initial decomposition of PBS and its interactions with PLA/CS-ADR, whereas the high-temperature peak is associated with the degradation of the main chain of the PLA matrix. This observation indicates that as the PBS content increases, the phase separation between the two polymers in the system becomes more pronounced, leading to reduced interfacial compatibility. Consequently, this results in a decrease in decomposition temperature, a reduction in the decomposition rate, and a more complex decomposition process [32].

From the TG curve, it can be observed that when the PBS content in the CS/PLA-ADR system ranges from 0% to 20%, the temperature at which the mass loss reaches 50% (T-50%) remains relatively stable, falling between 344 °C and 348 °C. Specifically, the T-50% values are as follows: CS/PLA-ADR at 347 °C, CS/PLA-ADR with 5% PBS at 348 °C, CS/PLA-ADR with 10% PBS at 344 °C, and both CS/PLA-ADR with 15% PBS and CS/PLA-ADR with 20% PBS at 346 °C. This stability suggests that the thermal properties of the material are relatively consistent within this range of PBS content. However, when the PBS content increases to 25%, the T-50% significantly decreases to 331 °C, indicating a substantial reduction in the thermal stability of the composite material at this composition. This phenomenon may be attributed to the occurrence of strong phase separation or a significant decline in interfacial compatibility between PBS and the PLA/CS-ADR matrix, which leads to earlier overall decomposition and an accelerated weight loss rate. Subsequently, when the PBS content increases to 30%, the T-50% rises again to 348 °C. This suggests that at higher PBS ratios, the microstructure or phase distribution of the system may undergo adjustments, resulting in decomposition characteristics of the PLA/CS-ADR and PBS phases that are reflected as a higher T-50% during thermal analysis [15]. Therefore, the introduction of PBS into the CS/PLA-ADR composite system does not produce a straightforward linear effect on thermal stability. Instead, it demonstrates an initial trend of relative stability, followed by a sharp decline at 25%, and then a partial recovery at 30% as the PBS content varies. This phenomenon illustrates the combined effects of compatibility, decomposition pathways, and potential phase separation behavior of PBS within the PLA/CS-ADR matrix at different ratios.

### 2.7. Effect of PBS Contents on Crystallization Properties of CS/PLA-ADR-PBS Composites

**Figure 7:**
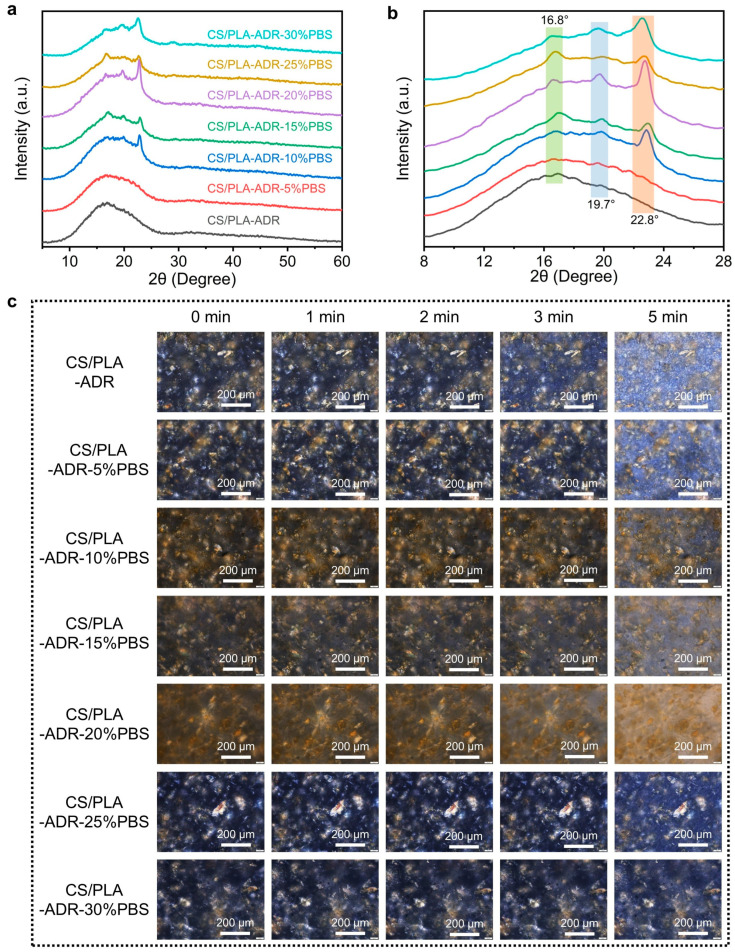
(**a**,**b**) XRD patterns and (**c**) POM photos of CS/PLA-ADR-PBS composites with different PBS contents.

The XRD patterns presented in Figure 7a,b indicate that the CS/PLA-ADR-PBS composites display distinct diffraction peaks at 2θ = 16.8°, 19.7°, and 22.8°. These peaks are primarily attributed to the crystalline structures of PLA and PBS. Specifically, the diffraction peak at 16.8° corresponds to the (110)/(200) crystal planes of the α-form crystal of PLA, while the peaks at 19.7° and 22.8° are mainly derived from the crystal plane diffraction of PBS [33]. With the increase in PBS content, the diffraction peaks at 19.7° and 22.8° become sharper or more intense, indicating that the crystallinity or grain size of PBS is maintained or enhanced in the system [34]. Meanwhile, the intensity and full width at half maximum of the PLA diffraction peak at 16.8° may change due to the interactions between PBS, ADR, and CS, reflecting the interference or promotion effects of the multi-component blend on the PLA crystallization process.

CS/PLA-ADR can be considered an integrated system that possesses both polarity and compatibilization functions. The intermolecular interactions between this system and PBS—such as hydrogen bonding, polar coupling, and dipole interactions—regulate the growth of PBS crystal planes and the distribution of crystalline regions to some extent. When PBS segments more readily form orderly arrangements within the CS/PLA-ADR system, the crystallization peaks of PBS become more pronounced. Conversely, if local regions exhibit phase separation or segmental interference, the crystallization processes of both PLA and PBS may be diminished, potentially leading to peak broadening. Additionally, the hydroxyl, amino, and other polar groups prevalent in CS, along with the epoxy, aromatic, and other functional structures potentially introduced by ADR, can significantly influence the crystallization kinetics of the composite material through interactions with the PBS main chain. This influence is evident in the XRD patterns, which exhibit changes in the position, full width at half maximum, and relative intensity of the diffraction peaks. Overall, the variations in the intensity and position of these diffraction peaks collectively illustrate the crystallization characteristics of PLA and PBS within the multi-component system, as well as the regulatory effects of CS and ADR on the material’s microstructure and crystallization behavior.

Figure 7c illustrates the crystallization behavior of composites with varying PBS content as observed under polarized optical microscopy. It is evident that as the PBS content increases, the crystallization regions of the CS/PLA-ADR-PBS composites initially decrease and then increase at the 0 min mark. The distribution of these regions transitions from relatively uniform to increasingly uneven. Furthermore, the crystallization rate of the composites gradually declines with the increasing PBS content. When the PBS content reaches 20%, the crystallization phenomenon becomes difficult to observe. However, as the PBS content continues to rise, crystallization becomes visible once again. At lower PBS concentrations, the chain extender ADR enables CS, PLA, and PBS to form a crosslinked network structure. This crosslinked network structure disrupts the crystalline regions of the composite material, making nucleation more difficult and slowing the crystal growth rate to the point where distinct crystallization phenomena become challenging to observe. However, when the PBS content reaches a certain threshold, the amount of ADR is insufficient to fully crosslink all the PBS with PLA and CS, resulting in some PBS remaining unbound within the composite. Since PBS is a flexible polymer with a high degree of crystallinity, its crystallization becomes evident under POM when more PBS aggregates together. Consequently, as the PBS content increases, the crystallinity and crystallization rate of the composite initially decrease before subsequently increasing.

### 2.8. Effect of PBS Contents on Thermomechanical Properties of CS/PLA-ADR-PBS Composites

**Figure 8:**
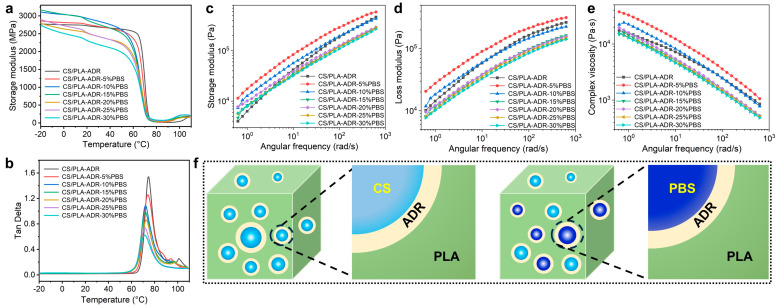
(**a**) Storage modulus, (**b**) loss angle curve, (**c**) storage modulus, (**d**) loss modulus, and (**e**) complex viscosity versus frequency curve of CS/PLA-ADR-PBS composites with different PBS contents, and (**f**) a mechanism diagram of PBS’s compatibilization and toughening for CS/PLA-ADR-PBS.

The effect of PBS content on the viscoelastic properties of the composites was investigated using dynamic mechanical analysis. The temperature-dependent curves of storage modulus and loss tangent (tan δ) are presented in Figure 8a and Figure 8b, respectively. From these figures, it is evident that the composites containing PBS exhibit a distinct two-stage decline in storage modulus. The first decline occurs within the temperature range of −20 to 100 °C, which is attributed to the relaxation motion of the side chains, known as β-relaxation. The second decline is observed in the temperature range of 20 to 80 °C and is associated with the glass transition (α-relaxation) [35].

As the PBS content increases within the temperature range of −20 to 70 °C, the overall trend of the storage modulus, despite some exceptions, initially rises and then falls, reaching a maximum value of approximately 3165 MPa at a PBS content of 15%. In the glass transition region, the onset temperature for the decline in modulus gradually shifts to lower temperatures as the PBS content increases. This observation is consistent with the decreasing trend of the glass transition temperature (T_g_) noted in DSC [24]. Additionally, the loss tangent values of the composites consistently decrease as the PBS content increases, dropping from 1.53 in the CS/PLA composite without PBS to 0.62 at a PBS content of 30%. This phenomenon is primarily attributed to the incorporation of flexible PBS segments, which also indicates an increase in the crystallinity of the composites. The narrowing of the loss tangent peak results from the increased crosslinking points between PLA and PBS, leading to a reduction in the original relaxation regions [36].

### 2.9. Effect of PBS Contents on Rheological Properties of CS/PLA-ADR-PBS Composites

As illustrated in Figure 8c, the storage modulus of CS/PLA-ADR-PBS composites demonstrates an overall trend of initially increasing and then decreasing with the rising PBS content. When the PBS content is low (e.g., 5% and 10%), the storage modulus of CS/PLA-ADR-PBS composites is significantly enhanced compared to that of CS/PLA-ADR composites. This enhancement is likely attributed to the formation of a crosslinked network structure among PBS, CS, and PLA facilitated by the chain extender ADR. The incorporation of PBS introduces highly flexible molecular chains into the composite system. At low PBS concentrations, its addition creates additional physical crosslinking points within the composite, thereby reinforcing the elastic network structure and consequently increasing the storage modulus [37]. However, when the PBS content increases to 15% or more, the storage modulus of CS/PLA-ADR-PBS composites begins to decline. This may be attributed to the excessive PBS disrupting the continuity of the elastic network structure within the composites, resulting in reduced compatibility and, consequently, diminished energy storage efficiency [38].

As illustrated in Figure 8d, the loss modulus of CS/PLA-ADR-PBS composites demonstrates an overall trend of initially increasing and then decreasing with the rising PBS content. At low PBS concentrations (e.g., 5%), the loss modulus of CS/PLA-ADR-PBS composites is significantly enhanced compared to that of CS/PLA-ADR composites. This enhancement is likely attributed to the capacity of low PBS content to form a crosslinked network with CS and PLA, resulting in improved interfacial bonding between the two phases. During the deformation process, the movement of molecular chain segments is enhanced, resulting in an increase in the loss modulus. However, as the content of PBS continues to rise, the loss modulus begins to decline. This decrease may be attributed to the excessive PBS weakening the interactions between molecular chains, allowing them to slide more freely, which subsequently reduces the loss modulus.

From the plot of complex viscosity versus frequency for the composites presented in Figure 8e, it is evident that the viscosity of CS/PLA-ADR-PBS composites initially increases and then decreases as the PBS content rises. When the PBS content is at 5%, the viscosity of the composites reaches its maximum value. However, as the PBS content continues to increase, the viscosity gradually declines. At lower levels of PBS, its incorporation creates additional physical crosslinking points within the composites, thereby enhancing the elastic network structure. This enhances the interactions between molecular chains, thereby increasing flow resistance and leading to a rise in viscosity. However, when the PBS content increases to 10% or more, the excessive PBS may function similarly to a lubricant. At this stage, the interactions between molecular chains are weakened, allowing the chains to slide more freely, which reduces the viscosity of the system.

### 2.10. Mechanism of CS/PLA-ADR Composite Capacitation and Toughening by PBS

As shown in Figure 8f, the CS/PLA-ADR-PBS composite system features epoxy groups from ADR that undergo ring-opening reactions with the amino, carboxyl, and hydroxyl groups of CS, PLA, and PBS. This reaction constructs a crosslinked network structure that significantly enhances the interfacial compatibility among the components. The incorporation of the PBS elastomer effectively introduces highly flexible molecular chains into the composite system. When subjected to external forces, the lower modulus of PBS creates a stress concentration point within the composite, which subsequently induces cavitation in the PBS. Cavitation can be classified into two types: debonding cavitation and internal cavitation. With the enhancement of interfacial bonding strength brought about by ADR, PBS is more susceptible to internal cavitation, resulting in greater energy dissipation. Additionally, cavitation promotes the formation of shear bands within the composites, which contributes to plastic deformation. This plastic deformation can effectively dissipate a substantial amount of energy through the sliding of molecular chains, thereby facilitating the toughening and compatibilization of the composites.

## 3. Methods and Materials

### 3.1. Experimental Materials

PLA granules (4032D, density 1.24 g/cm^3^, melting point 165 °C, melting index 1.25 g/m^3^) were purchased from NatureWorks LLC (Minnetonka, MN, USA). CS (degree of deacetylation ≥95%) was obtained from Shanghai Aladdin Biochemical Technology Co., Ltd. (Shanghai, China). The chain extender ADR (4468, Mw 7250 g/mol) was sourced from BASF (Düsseldorf, Germany), and PBS (TH803S) was acquired from Xinjiang Blue Ridge Tunhe Chemical Industry Co., Ltd (Changji, China).

### 3.2. Preparation of CS/PLA-ADR-PBS Composites

**Figure 9:**
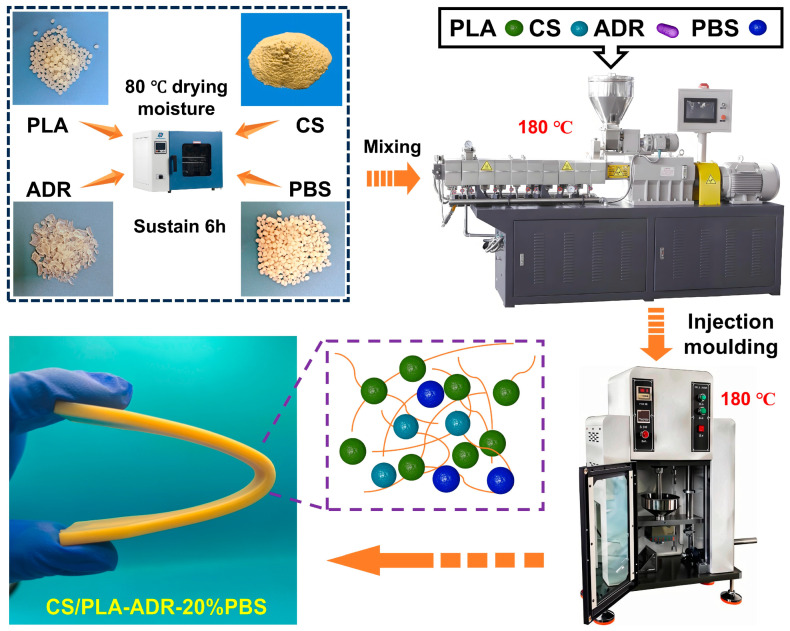
Illustration of CS/PLA-ADR-PBS composite materials preparation.

CS, PLA, and PBS were dried in a vacuum oven at 80 °C for 6 h to eliminate moisture. The materials CS, PLA, PBS, and ADR were blended according to the ratios specified in Table 2 using a twin-screw extruder (SHJ-20, Nanjing Jiente Machine Point Co. Ltd., Nanjing, China). The temperature settings from the die to the feed zone were maintained at 135 °C, 165 °C, 180 °C, 175 °C, and 135 °C, respectively, to produce CS/PLA-ADR-PBS composites. The blended CS/PLA-ADR-PBS composites were then crushed into granules using a multifunctional crusher. Injection molding was conducted with a micro-injection molding machine under conditions of 180 °C, 0.8 MPa pressure, and a heating time of 300 s to create dumbbell-shaped and strip-shaped samples (a preparation illustration is presented in Figure 9).

### 3.3. Characterization

Chemical structure: The changes in the chemical structure of the composites were characterized using a Fourier Transform Infrared (FTIR) spectrometer (Tensor II, Bruker, Hong Kong). The wavelength range was set from 500 to 4000 cm⁻¹, with a resolution of 4 cm⁻¹ and 32 scans.

Mechanical performance: The mechanical properties of the materials were evaluated at room temperature using an electronic universal testing machine (CMT-5504, SANS Testing Machine, Shenzhen, China). Both tensile and three-point bending tests were conducted at a speed of 2 mm/min, in accordance with national standards GB/T 1040.1-2018 and GB/T 9341-2008 [39,40]. The tensile test specimens were shaped like dumbbells, with dimensions of 75 mm × 5 mm × 2 mm and a cross-sectional width of 5 mm. The three-point bending test specimens measured 80 mm × 10 mm × 4 mm. The unnotched impact strength of the materials was evaluated at room temperature using an XJC-50 simply supported beam impact testing machine in accordance with the national standard GB/T 1043.1-2008 [41], utilizing a pendulum with a capacity of 2 J. The impact test specimens were prepared using molds measuring 80 mm × 10 mm × 4 mm. Prior to conducting the tensile, bending, and impact tests, the dimensions of each specimen were measured with a vernier caliper. A minimum of five tests were performed for each sample composition, and the results were averaged.

Morphology recording: The microstructure of the samples was examined using a scanning electron microscope (QUANTA 220, FEI Company, Hillsboro, OH, USA) at an accelerating voltage of 10 kV.

Water absorption (WA) test: The test was conducted in accordance with GB/T 1034-2008 [42]. Specimens measuring 10 mm × 10 mm × 4 mm were vacuum-dried at 60 °C for 12 h and then immediately weighed, recorded as $W_{a}$. They were subsequently immersed in a beaker of ultrapure water at room temperature. During the test, the specimens were removed every 12 h, and the surface water was blotted with filter paper before weighing. This process continued until the mass stabilized, recorded as $W_{b}$. The water absorption was calculated using Equation (1).

(1) $${\mathrm{WA}=(W}_{b}{-W}_{a}{)/W}_{a}\times 100\%$$

Differential scanning calorimetry analysis: The thermal properties of the samples were tested using a differential scanning calorimeter (DSC, 214 Polyma, NETZSCH Instruments, Germany). The samples were heated from 25 °C to 210 °C at a rate of 10 °C/min. The crystallinity of the samples was calculated using Equation (2).

(2) $$X_{c}=\frac{{\Delta H}_{m}-{\Delta H}_{c}}{{\Delta H}_{f}\times W_{\mathrm{PLA}}}$$

In the formula, $X_{c}$ represents the crystallinity of the sample; ${\Delta H}_{m}$ represents the melting enthalpy of the sample; ${\Delta H}_{c}$ represents the cold crystallization enthalpy of the sample; ${\Delta H}_{f}$ represents the melting enthalpy of PLA when fully crystallized, which is 93.7 J/g [43]; and $W_{\mathrm{PLA}}$ represents the mass fraction of PLA in the sample.

Thermal stability: Thermogravimetric analysis (TGA) of the samples was conducted using a simultaneous thermal analyzer (209F3, NETZSCH, Selb, Germany). The test temperature range was 25–600 °C, with a heating rate of 10 °C/min and an argon flow rate of 30 mL/min.

Crystalline structure: The crystalline structure of the materials was characterized using an X-ray diffractometer (D/max 220, Rigaku Corporation, Tokyo, Japan). The scanning rate was 5°/min, and the scanning range was 5–50°.

Observation of the crystal nucleation and growth process: The spherulite nucleation and growth process of the samples was observed using a polarized optical microscope (BX53M, OLYMPUS, Tokyo, Japan). The samples were pressed into thin films and placed in an observation dish on a hot stage. The temperature was raised to 180 °C to melt the samples and eliminate any thermal history, and then rapidly cooled to 120 °C to observe the crystallization process.

Dynamic mechanical thermal analysis: This analysis was performed using a dynamic mechanical analyzer (242 E, NETZSCH, Germany) in a three-point bending mode. The composite samples measured 45 mm in length, 5 mm in width, and 2 mm in thickness. The heating rate was set at 5 °C/min, with a frequency of 5 Hz, and the temperature was increased from −20 °C to 150 °C.

Rheological performance: The material’s performance was analyzed using a rotational rheometer at 180 °C with a 25 mm parallel plate fixture (gap of 2 mm). A frequency sweep ranging from 0.1 to 100 rad/s was conducted at a strain amplitude of 0.1% to obtain the melt dynamic performance data.

## 4. Conclusions

In conclusion, in this study, biodegradable PBS elastomer was utilized as a compatibilizer and incorporated into the CS/PLA-ADR system to create CS/PLA-ADR-PBS composites. The introduction of PBS, through the synergistic effects of flexible chain segments and a crosslinked network, significantly altered the mechanical properties of the CS/PLA-ADR composites. The elongation at break reached a peak value of 159.02% at 20% PBS, representing an increase of approximately 123% compared to the control group. SEM analysis revealed that PBS enhanced toughness by forming a co-continuous phase and a tear fiber structure. However, excessive addition of PBS (greater than 20%) resulted in phase separation, with the sea–island structure accounting for up to 35%, leading to a decrease in interfacial bonding strength. DMA indicates that the appropriate addition of PBS can enhance the elastic network through physical crosslinking. Furthermore, the introduction of PBS flexible chain segments reduces the frictional energy dissipation of molecular segments. Considering the mechanical properties, compatibility, crystallization characteristics, thermal properties, and rheological properties, the CS/PLA-ADR-PBS composite with a PBS content of 20% exhibits optimal overall performance. At this concentration, PBS is uniformly dispersed within the PLA matrix, with strong interfacial bonding, forming a co-continuous phase structure that effectively enhances energy absorption capacity. This work significantly enhanced the toughness and elongation at break of PLA composites, thereby broadening the related applications in packaging and film bag fields. It also provides a reference for the toughening research of other PLA composites, thereby expanding the application field of PLA in biodegradable materials.

## Figures and Tables

**Table 1:** DSC parameters of CS/PLA-ADR-PBS composites with different PBS contents.

Formulation	T_g_ (°C)	T_cc_ (°C)	T_m_ (°C)	ΔH_m_ (J/g)	X_c_ (%)
CS/PLA-ADR	63.63	110.8	168.6	3.05	3.39
CS/PLA-ADR-5%PBS	62.01	109.17	166.68	1.07	1.12
CS/PLA-ADR-10%PBS	61.50	109.45	167.00	3.33	3.29
CS/PLA-ADR-15%PBS	61.10	109.32	166.90	3.58	3.36
CS/PLA-ADR-20%PBS	61.20	109.19	166.83	6.776	6.03
CS/PLA-ADR-25%PBS	60.26	110.67	166.71	8.349	7.08
CS/PLA-ADR-30%PBS	60.83	109.48	166.62	12.259	9.93

**Table 2:** Proportions of the components of the composite.

Sample	CS (wt%)	PLA + PBS (wt%)	PLA /(PLA + PBS)	PBS /(PLA + PBS)	ADR (%)
CS/PLA-ADR-5%PBS	4	96	95%	5%	1.5
CS/PLA-ADR-10%PBS	4	96	90%	10%	1.5
CS/PLA-ADR-15%PBS	4	96	85%	15%	1.5
CS/PLA-ADR-20%PBS	4	96	80%	20%	1.5
CS/PLA-ADR-25%PBS	4	96	75%	25%	1.5
CS/PLA-ADR-30%PBS	4	96	70%	30%	1.5

## Data Availability

Data will be made available on request.
